# The Anti-Inflammatory Activity of *Viscum album*

**DOI:** 10.3390/plants12071460

**Published:** 2023-03-27

**Authors:** Marcello Nicoletti

**Affiliations:** Department of Environmental Biology, Sapienza University of Rome, 00185 Rome, Italy; marcello.nicoletti@uniroma1.it

**Keywords:** *Viscum album*, cancer, inflammation, immunity, antioxidant, microProteins, viscotoxins, lectins, flavonoids

## Abstract

The therapeutic story of European mistletoe (*Viscum album* L.) presents a seesawing profile. In ancient times, this hemiparasitic plant was considered a panacea and even to be endowed with exceptional beneficial properties. In more recent times, despite its multiple uses in traditional medicines, some parts of the plant, in particular the berries, were considered poisonous and dangerous, including concerns of cytotoxicity, which spread serious suspicion on its medicinal utility. However, since the last century, medical interest in mistletoe has come back in force due to its utilization in clinical cancer treatments, based on its selective action on tumor cells. In Central Europe, the hydro-alcoholic extracts of European mistletoe register a relevant and continuous utilization in anthroposophic medicine, which is a holistic system that includes the utilization of phytomedicinal substances. In Switzerland and Germany, most physicians and patients use these products as complementary therapy in oncological treatments. However, despite its increasing use in this field, the results of mistletoe’s use are not always convincing, and other aspects have appeared. Nowadays, products that contain mistletoe are utilized in several fields, including diet, phytotherapy, veterinary medicine and homeopathy, but in particular in cancer therapies as coadjuvant factors, in consideration of several positive effects including effects in the improvement of quality-of-life conditions and reinforcement of the immune system. In this review, based on the understanding of the association between cancer and inflammation, we propose a relationship between these recent uses of mistletoe, based on its antioxidant properties, which are supported by phytochemical and pharmacological data. The unicity of mistletoe metabolism, which is a direct consequence of its hemiparasitism, is utilized as a key interpretation element to explain its biological properties and steer its consequent therapeutic uses.

## 1. Introduction

Medical treatment of diseases is an open work in progress, being dependent not only on scientific evidence, but also on cultural, social, political, economic and other temporary influences. The history of medicine is full of more or less radical changes and difficulties in acceptance of revision in medical drugs and their utilization. This is particularly evident in the therapeutic use of medicinal plants. The controversial medical utilization of mistletoe throughout the centuries is here reported as a case study, focusing on the recent relation between inflammation and cancer, including effects on the immune system and general living condition. 

### 1.1. Mistletoe (Viscum album L.), a Special Plant

*Viscum album* L. (Santalaceae, formerly Viscaceae or Loranthaceae) is the most common species of its genus (c. 450 species) [1], mainly present in Europe and Asia and commonly known as European mistletoe or simply mistletoe [2]. *Viscum* plants are an exceptional example of diversity in dicotyledonous angiosperms, though in their general appearance and morphology they are very similar to many other shrubs present in temperate flora [3]. It is an evergreen woody shrub, with stems 30–100 centimetres long characterized by abundant dichotomous branching. The entirety of the leathery strap-shaped leaves are yellowish-green in colour. This species is dioecious with inconspicuous, yellowish-green flowers, whereas the fruits are typical, as opalescent globose berries, containing a very sticky, glutinous pulp embedding one (very rarely several) seed [4].

Unicity of *Viscum* spp. concerns the physiology, since they are epiphytic and obligate hemiparasitic plants. They are completely wrapped inside the crowns of broad-leaves of the host, where they can be found by looking carefully. The obligate hosts are several deciduous and needle trees, such as apple, linden, hawthorn, oak and poplar, but also pines and firs, as well as shrubs, such as hawthorn, whereas mistletoe cannot be found in *Fagus* or *Platanus* species. However, the Viscum’s parasitism is dependent on the available flora, since in Nigeria and Ghana, it can be found on cocoa, kola and cashew trees [5].

In these regions, the mistletoe is evident as a denser prominent globose silhouette up to 1–2 m in diameter, distinguished by the rounded shape and deeper green colouration of the abundant leaves inside the crown of the tree host [6]. This is a consequence of its aerial hemiparasitism, consisting of the metamorphosis of the root system, which is converted into an endophytic haustorial organ structure that is able to enter inside the host’s tissue at the xylem level and suck water and nutritional substances contained in the sap [7]. This allows the plant to avoid any ground contact, and it is the cause of many anomalies. In fact, mistletoe is a common name for several independent lineages descended from sandalwood. As well, sandalwood species belonging to the same family Santalaceae are hemiparasites, but they insert sinker haustoria inside the host roots [8,9]. Unlike those ancestors, mistletoes do so in the sky. Especially in weather conditions with water stress, due to competition with the host tree, the parasitism of *V. album* can cause problems in the growth and vitality of the host [10,11]. However, under usual and favourable conditions, host trees are only moderately affected, and often both can coexist well [12,13].

Evidence of Viscum parasitism is hidden in its metabolism, characterized by an extraordinary life cycle and unique biochemical properties, based on a genome that is extremely large, estimated to be 600 times the genome of *Arabidopsis thaliana* [14], which is considered the model plant in angiosperms. In addition to reported distinct feature differences in the photosynthesis process, consisting of the different ultrastructures of chloroplasts and lower amounts of chlorophylls [15], attention has been focused on the particularities of the respiratory system. European mistletoe is the only known multicellular organism able to survive without the mitochondrial NADH dehydrogenase complex of the respiratory chain, which catalyses the electron transport chain essential for the production of the energy necessary to fuel cell metabolism. Its respiratory system, due to its restricted capacities to generate mitochondrial ATP, is believed to survive by relying to a great extent on obtaining energy provided by its host tree and on alternative respiratory enzymes [16,17].

Furthermore, studies on the photosynthetic apparatus of *V. album* [18] evidenced relevant differences in mitochondria and thylakoids in comparison with other angiosperms, including the corresponding changes in the genome due to adaptation [19]. In this case, it is possible to assume that *V. album*’s metabolic apparatus is tailored to interact with the cells of other organisms. However, these are not the only surprises. Viscum plants possess the ability to parasitise another mistletoe that has already parasitised a host tree. This rare facultative tripartite network is a form of special association named hyperparasitism. When mistletoe undergoes an obligate association with another species of mistletoe, it is better called epiparasitism. In the case where the parasite is a member of the same species, this is named autoparasitism [20]. The lesson is that the more we investigate and study the biological phenomenon called parasitism [21], the more we have to learn, which means our knowledge must be continually updated. This should be considered in all aspects concerning *V. album*, including its uses.

Among the evidences of this situation, the impressive variability and adaptability of *V. album* merit special consideration [22]. The total preference for evergreen hosts allows the plant to photosynthesise throughout the year and at low temperatures below the freezing point, although at a rate lower than the host’s assimilation organs. For the reasons already reported, the *V. album* species is subdivided into several subspecies and varieties, which are specialised according to the host tree or shrub [23]. Different from the common action, *V. album* rarely grows on oak trees, wherein it is substituted by another hemiparasite, *Loranthus europeus* Jacq. (Loranthaceae), the oak tree or yellow mistletoe. The two species are quite similar in shape but very different in chemical constitution, as evidenced by the metabolomic HPTLC analysis of bud extracts [24]. Therefore, in this case, the exact botanical identification, made by a specialist in botany, is necessary. 

The relationship between mistletoe and its host, besides behaviour, primary metabolism and ecology, deeply affects the secondary metabolism as well [25,26]. Many studies evidenced a relevant interchange and influx of material and information between the metabolisms of the parasite and the host [27,28,29,30], as evidenced also by the characteristics of the haustorial system and the parasitism of selected aerial parts of the host [31]. As in other investigated cases of parasitism, the conclusion is that in these cases, parasite and host partly compart metabolism, giving rise to a fused complex dynamic system, such as those named superorganisms. A superorganism is a successful and efficient organization of different living organisms. They are everywhere, for instance plentifully in the human body, and they are the fuel of evolution, in contrast with the negative effects of selection struggle competition. The plant no has direct link to the substratum, and the ground properties cannot influence it directly. The host has an intermediate role, but both organisms have to face habitat challenges together. 

Phytochemical studies have reported the typical large quantity of natural products in angiosperms [32]: terpenoids, including steroidal and acid triterpenes, glycosides of different types, phenols in a wide range of types, phenylpropanoids, coumarins, as well as several kinds of amino acids, minerals and others. As usual, most of them are already known constituents, but also new compounds were identified, including the absence of common alkaloids in favour of a small but relevant presence of a new class of unusual aminoalkaloids [33]. 

The highly complex chemical composition, fully confirmed by HPTLC analysis [24], and biological activity of mistletoe are the result of its special metabolism, which is the consequence of a mixture of biotic effects (mainly host tree) and abiotic environmental characteristics, influencing both parasite and host, but in a different way. A complete report of *V. album* secondary metabolites until 2000 can be found in [34]. Their identifications were obtained by the usual phytochemical techniques, but much more information is being obtained by further research based on metabolomics approaches [35,36]. The metabolic approach on Romanian *V. album* evidenced the predominance of flavonoids, amino acids and peptides and terpenoids, representing about 60% of all metabolites identified; however, among them, flavonoids were predominant, accounting for more than 30% of the total identified constituents [37]. Among these compounds, the utilization of over 250 primary and secondary in medicine, present in leaves, shoots and berries, has been reported [38]. Variations in quantity and quality were also observed for different compounds [39,40], including triterpenes [41] and antioxidants [42,43]. The highest levels of total phenolics, flavonoids and antioxidant effects of *V*. *album* were observed in the leaf extract compared with fruit and seed [44,45].

Special attention must be paid to specific oligoproteins in consideration of their biological activity [46]. The foliage and fruits of *V. album*, as in other related species, contain low-molecular-mass proteins of the thionin type, designated as viscotoxins, as well as characteristic lectins, named viscolectins, both of which are able to contribute to its defence system and can be found in related extracts and products. These classes of Viscum microProteins have received considerable attention because of their contribution to cytotoxic and immune-stimulating effects of the mistletoe extracts used in medicine, as later discussed, including their mechanism of action [47,48,49].

Several isoforms of viscotoxins have been reported, with A1, A2, A3 and B considered the predominant ones and usually measured in quality controls. Viscotoxin A3 is usually the predominant isoform [50,51,52,53,54] and also the most active, as confirmed by comparative membrane interactions [55]. However, the total proteome of thionins is much more complicated, and includes viscumin, reported as very active but present in very low quantity [56,57,58,59]. Viscumin is reported able to participate in in vitro activity, including inhibition of protein synthesis in a cell-free system, and pertains to the pull of thionins, and also is reported as responsible for cytotoxicity [60]. 

Lectins are well-known for their capacity of linkage to the sugar component of membrane cells (the name derives from the Latin word *lego*, meaning “to link”). The biochemistry and structures of three types of *V. album* lectins were studied and characterized, termed mistletoelectins I, II and III (MLI, MLII and MLIII) [61,62,63], evidencing their role as precursors to proteins and the structures based on two chains, post-translationally cleaved into an alpha and a beta chain. The two chains are stabilized by linkage via disulfide bridges [64]. The beta chain, in accordance with the lectin activity, specifically binds to sugar residues of membrane proteins, inducing its endocytic uptake by target cells. The alpha subunit has RNA glycosidase activity. The consequence is possible damage to the involved cells, thereby inactivating the ribosome and inducing apoptosis [65]. However, further studies that focused on clarifying this aspect have shown that “direct apoptosis induction by mistletoe lectins occurs only after uptake of the molecules into the cell due to the action of the ribosome inactivating A-chain”, meaning there is no evidence for cytotoxic effects caused by isolated A- and B-chains [66,67].

Considering that most of the organic matter of any living organism is made of proteins (c. 80%), the content in marketed products of European mistletoe, used as food or for therapeutic uses, could appear irrelevant, being in the order of few mg/g of dry material for viscotoxins and even ng/g for viscolectins, but they have attracted great interest for their specific biological activity. However, most of the metabolome is made by large high-molecular weight proteins inside the structural network of the organism, whereas microProteins, are small, less than 20 KDa, and contain a single domain [68,69]. This domain allows plant microProteins to interact with other compatible domains of evolutionary-related proteins and perform specific key physiological effects in several organisms. In particular, microProteins are recently under deep investigation, in consideration of their role in plant development [69,70]. Viscotoxins are cationic cysteine-rich proteins composed of only 45 *amino acids* and with a molecular weight less than 5 KDa [67].

The presence and content of Viscum microProteins, and therefore their content in related marketed products, is subject to several variations. Although the foliage (meaning leaves, little branches and shoots when present) content is higher than the fruit one, again the percentage is subject to high variation in relation to the subspecies, the host, the season and the ecological conditions [70], assigning importance to the harvesting time. It is expected that the pharmacological activities of mistletoe extracts, such as cytotoxicity and induction of apoptosis in cancer cells, vary accordingly. 

The anticyclic timing of leaf senescence and abscission in mistletoe, which occurs in the summer, has been observed, when on the contrary the metabolic activity in the host has reached its maximum [71]. It is possible that viscotoxins play a significant role, since in that period they only degrade, whereas most of the remaining leaf proteins are lost during abscission, and the changes in viscotoxin content are similar to changes in the concentration of the corresponding mRNA; however, shortly before the onset of leaf senescence, the mRNA for viscotoxin disappears from the leaves [72]. These are additional signs of the exceptional biological activity of the microProteins of *V. album*. 

Further information came from the study of viscotoxins production. In 2022, Schroder et al. [73] deeply examined the genome of *V. album*, reporting new insights into the metabolism and molecular biology of the plant, including the biosynthesis of lectins and viscotoxins. Considering the paradox of the extremely large genome and the low dimension of produced proteins, they focused their attention on the analysis of the sequencing of the *V. album* gene space (defined as the space including and surrounding genic regions, encompassing coding as well as 50 and 30 non-coding regions). The analysis of the mitochondrial genome allowed them to evidence that the *V. album* gene space includes a transcript encoding the enzyme L-galactono-1,4-lactone dehydrogenase (GLDH). GLDH is localized in the mitochondrial intermembrane space and catalyses the terminal step of the mitochondrial ascorbate biosynthesis pathway [74]. This information could be related to the selective action of *V. album* extracts on cancer cells, as further discussed. The paper is an example how the introduction of new potent analytical techniques and tools, such as hyphenated approaches including HPLC-MS/MS [75,76], can be fundamental in obtaining reinterpretations of biological effects, so far hastily catalogued. Several authors also argue the necessity of opening the consideration of the constituents considered responsible of the activity of *V. album* extracts, including the toxic effects, and avoiding focusing the total attention on microProteins [77]. 

### 1.2. The Controversial Medical Story of Mistletoe

According to Luther and Becker, European mistletoe has been studied for more than 2000 years, in particular for its medicinal uses [78]. Some features remained practically the same through the centuries, whereas other ones changed [79]. Since ancient times, European mistletoe’s therapeutic properties, such as antidiabetic, analgesic, anti-inflammatory, anti-arrhythmia and hypotensive properties, were already known in popular and traditional medicines of Europe, Asia and Africa, and are still well present in the medicines of several countries [80,81,82,83,84,85]. In the Occident, as many other medicinal plants, after the old periods of practice in popular medicine, it suffered a long period of obscurity and prevention of use, until a recent renaissance [86].

Mistletoe is generally known as a talisman of good fortune and prosperity. In fact, it has long been considered a pagan religious symbol of prosperity and was believed to have magical properties. The use of mistletoe in ritual form is mainly related to traditions of Celtic druids, the ancient civilisation of people living on the British Isles, in what is now Ireland and Scotland [87]. The Roman historian Plinius Secundum (23–79 AD) reported that druids mainly used mistletoe, collected on the oak, as a panacea (“Viscum” which should mean “able to cure any disease”), and considered it to be a sacred plant [88]. However, Greeks and Romans did not believe the mistletoe’s healing powers, as with everything coming from “Barbarians”, as reported by Plinius: “They believe that mistletoe, taken in potion, gives the ability to reproduce to any sterile animal, and that it is a remedy against all poisons: so great is the devotion that certain peoples turn to things mostly of no importance” (translated by the author). On the contrary, in Viking sagas, the plant is related to the death of Baidr (or Baldur), grandson of the Norse god Thor, stabbed and killed by an arrow made from the wood of a mistletoe plant [89]. In any case, the conquest of Gaul by Julius Caesar erased any Celtic cultural heritage and obliterated the ancient knowledge of druids, including the complex procedures to select, collect and use mistletoe. Consequently, the healing properties of mistletoe were obscured for centuries, whereas the poisonous effects of eating berries became dominant. The original name changed to mean a substance that is gooey, adhesive and gluey, as the pulp of the berry once used to trap birds; however, in the plant, the sticky pulp, made by hemicelluloses, is necessary for the dissemination made by birds, since the seeds do not germinate in soil and in this way stably attach themselves to the bark branches of the tree, making possible the growth of new plants [89,90]. 

However, reading carefully the documents of scholars through the Middle Ages, including the Dioscorides De Materia Medica, the healing properties of European mistletoe preparations are always reported. Hippocrates recommended its use to treat diseases of the spleen and complaints associated with menstruation. Paracelsus described its benefits in the treatment for epilepsy. Hildegard von Bingen considered mistletoe as useful in the treatment of diseases of the spleen and liver. Moreover, ethnobotanical studies evidence a wide range of continuous uses in the popular medicine of every part of the world wherein plants of the *Viscum* genus are present, without evidence of very rare adverse effects [91]. 

As a matter of fact, for a long time in the textbooks of botany and pharmacognosy, mistletoe was considered an extravagantly dangerous invention of nature, and by the end of the nineteenth century, mistletoe was rejected by scientists, and retreated as a folklore remedy, although mistletoe was also commonly used in the medicines of other parts of the world, including Japan, Israel and several African countries [92,93,94]. This remained the case until a century ago, when something changed [95]. 

In 1920, Rudolf Steiner introduced *V. album* in cancer treatment as a drug extract, obtained by a complicated manufacturing process, combining sap from mistletoe harvested in winter and summer [86]. Rudolf Steiner (1861–1925) was an Austrian scientist and philosopher considered the founder of anthroposophic medicine, together with Ita Wegman [96]. Thereafter, the use of Viscum extracts in medicine increased, in particular in the countries of Central Europe [97]. During the last years, the antitumor activity of *V. album* preparations has been reported and checked in many clinical reports from 1919, with potential effectiveness in the treatment of neuroblastoma [98] and other cancer cases [99,100]. Viscum preparation was used in clinical combined therapy of advanced metastatic cancers [101,102,103,104,105,106,107,108,109], whose effects were mainly attributed to viscotoxins and viscolectins.

The first concern of physicians was the evidence of absence of toxic and adverse effects, meaning safety in the treatment. The results were encouraging [110,111], but, in continuing the therapies, the attention of physicians working in the hospitals was attracted by other effects, concerning the improvement of the quality of life of patients under conventional and palliative oncological treatments [112,113,114], registering the reduction of serious collateral effects of chemotherapy in treatment of several tumor cases, including breast, ovarian, non-small cell lung cancers and, on the contrary, the evidences of beneficial effects [115,116] were increasing and the attention shifted, changing again the perspective.

Nowadays, despite the high number of reported positive cases, including better survival [117,118,119,120,121,122,123,124], the cure for cancer using *V. album* is considered not totally valid and its effectiveness is under discussion [125,126,127]. However, it has to be admitted and added that at the present there are not totally sure and effective medical solutions to free humanity from cancer. However, the observation about the use of *V. album* commercially available products as adjuvants appeared significant in number and quality, and related to the improvement of the immunoadjuvanten and immunomodulating properties in a dose-dependent way [128,129,130,131,132,133], influencing the dendritic cells and showing other effects [134].

### 1.3. The Anti-Inflammatory Properties of Mistletoe

The utilization of *V. album* in cancer therapy was reinforced by several studies, which evidenced the character of the cytotoxic action of microProteins of *V. album* by several pharmacologic tests selectively directed at cancer cells, utilizing the capacity to link to membrane sugar and clarifying the absence of severe adverse effects during the treatments [135]. On the other hand, the positive effects during the treatments need to be explained and assigned to some other activities of Viscum products, including their anti-inflammatory activities.

The anti-inflammatory properties of *V. album* preparations and marketed products have been reported. The activity was supported by evidence on effects on many factors related to the inflammation process. In particular, the constituents of *V. album* were investigated for COX-2 inhibitory effect, selective inhibition of cytokine-induced expression of cyclooxygenase, mediated PGE2 and COX-2 inhibition implicating COX-2 mRNA destabilization [136,137,138].

In many papers, the anti-inflammatory activity was reported in connection with other activities, including the antioxidant [139,140,141,142,143,144] and anticancer [145,146,147]. Among the constituents of *V. album*, besides microProteins [148], other constituents, i.e., phenols and in particular flavonoids, have been considered responsible for this activity [36,143,149,150], in consideration of their well-known anti-ROS and radical-scavenger properties [151], and of the isolation of new flavonoids in *V. album* [152,153]. Most of the studies amplified the research to other constituents to evidence how they may co-operate in the activity [149,150,151,152,153,154,155,156], including new phenolic compounds isolated from the whole plant [157]. In several papers, more activities are considered on the basis of a connection made between inflammation and many related pathologies [157,158,159,160,161,162,163,164,165,166,167,168,169,170]. In the medical point of view, inflammation is divided into acute and chronic inflammation, according to causes, symptoms and medical treatment. Another important aspect is the age of the patient and the different immunological tasks. The aim of the treatment of the acute inflammation is focused on elimination of responsible agents, such as antigens, viruses, bacteria, etc., whereas in chronic inflammation the catabolic and destructive aspects are predominant, and therefore therapy is focused on restoring the homeostasis of the organism. 

In Table 1, the reported studies on anti-inflammatory activity of *V. album* extracts and derived products (VA) are reported. Due to the paramount utilization of VA products in oncology and connected palliative and adjuvant therapies, most of the studies are dedicated to supporting the efficacy of these treatments. As a consequence, the extracts, or directly the products, were utilized. In most cases, further investigations concerning the analysis of active constituents are necessary. However, several reports evidence in deep detail the related mechanisms and factors. In consideration of the related activity, in Table 2 the reports concerning the antioxidant activity are reported. Again, most of the studies concern the registration of the activity, including radical-scavenger properties, without the necessary analysis of the substances responsible of the properties, insisting on total phenolic content and obsolete tests. This is the consequence of a limited approach in the study of medicinal plants, wherein phytochemists and pharmacologists independently conduct their work, whereas a complete interaction is necessary. The metabolomics approach is causing a revolution in the phytochemical analysis and pharmacology should change, in accordance. The future of phytomedicines relies on this challenge.

Most of the studies concerning the anti-inflammatory activity of *V. album* may be evaluated on the hypothesis that the inflammation is basically an automatic reaction of the body to any kind of deviation from the homeostatic state [173]. As consequence of an external attack, such as an infection or an injury, or an internal dysfunction, such as an auto-immune or tumor disease, inflammation is in charge of activating the physiological processes indispensable to restart or maintain the homeostasis. However, chronic inflammation is a common accomplice of disease. Therefore, inflammation is on one side related to the cause and on the other side to the connected reaction to the disease, either a signal, a replay or a pathologic state. Nowadays, the medical treatments are dedicated to the signals, meaning the boring and debilitating state, and consist mainly in removing the symptoms, meanwhile waiting for the immune system to do its job and restore the homeostatic condition [174,175]. In many cases, as in most infections, this coupling works perfectly in a few days, otherwise the magic pill results are not sufficient and another approach is necessary, considering all the required aspects. In cases of research on the antioxidant and anti-inflammatory activities of *V. album*, the tendency is in favour of connecting inflammation to the pathology and considering every possible effect. To obtain this result, the utilization of mixes of active substances is considered necessary, due to the multiple targets. In accordance with the phytocomplex approach, any plant extract contains many different substances with different activity. In this regard, the antibacterial activity must also be considered since bacteria are also one of the intrinsic and extrinsic factors that can cause inflammation cancer, and the antimicrobial activity has been connected with antitumor properties [176,177,178,179,180,181]. The utilization of *V. album* preparations in the treatment of cancer and anti-inflammatory activity can now be used as a case study. 

### 1.4. Inflammation and Cancer

In war, the first immediate measure is to kill as many enemies as possible with the best available weapon, without concern about the consequences. Consequences always come. In the report of February 2022, WHO stated that cancer is a leading cause of death worldwide, accounting for nearly 10 million deaths in 2020, or nearly one in six deaths. Cancer is considered the second main cause of death, after cardiovascular diseases [182]. For a long time, the main therapies of cancer have been directed at the target, as selectively as possible, consisting in destroying any cancer cells by surgery, chemotherapy, radiation therapy, hormone therapy and/or immunotherapy. A key problem concerns the selectivity. Most of conventional cancer treatments suffer from severe off-target effects, as a consequence of sharing critical facets of cells, generally targeted by all rapidly proliferating capacity. The WHO also stressed the importance of improving the patient’s quality of life as a fundamental goal, which can be achieved by support for the patient’s physical, psychosocial and spiritual well-being, including palliative care in terminal stages of cancer in connection with other drugs. 

It is well-known that cancer treatments can be devastating for the patient, who absolutely needs support, to be able to face the impact of the disease and the cure. Furthermore, the immune system must be in a condition to play its fundamental role. Consequently, the so-called cocktails of drugs are increasingly utilized to treat cancer, as well as other impacting diseases. New therapeutic agents are urgently required to increase selectivity, help the immune system, reduce side-effects and support general health conditions. 

The relation between cancer and inflammation has been widely demonstrated. As recall by Pushpa Hegde, “inflammation and cancer dance together towards disaster”. Inflammation is considered a hallmark of cancer, and related to the spread of the disease within the body and the resistance of cancer cells to the treatment. Chronic inflammation is closely related to oxidative stress and immunosuppression [183,184,185,186,187,188,189]. 

Cancer development is dependent on inflammation, since when cancer causes necrosis of healthy cells, they release cell contents into the environment, triggering the release of pro-inflammatory mediators [190]. Further, the responses to therapy are regulated by inflammation, which either promotes or suppresses cancer progression [191], together with the general life conditions and the ability to promote an immune response [192].

Among the properties of the neoplastic cells to resist cancer treatment, the altered lipid metabolism, and consequently the abnormal cell membrane composition, plays a central role. Changes are necessary to the accumulation of energetic material, which could be used for a higher proliferation rate, obtained by the modulation of certain enzymes [193]. Consequently, some biophysical properties of cell membranes, namely, the composition of cell membranes in tumor cells, leads to cancer biomarkers, a higher resistance to chemotherapy and finally to the response of the immune system. Particularly interesting is the significance of the cell membrane content in the modulation of metastasis [194]. Clinical studies show novel therapeutic options concerning the modulation of cell membranes in oncology [195,196].

To understand the possible role of antioxidant and anti-inflammation of constituents of *V. album* in the fight against cancer and other diseases, it is necessary to inter-cross the information about the four items so far considered: cancer, inflammation, immune system and metabolome. 

## 2. Conclusions

Although further data trials need to be performed, the examination of clinical and pharmacological evidence on the majority of oncological patients so far allows us to consider European mistletoe extracts able to cause various anticancer achievements [197,198,199,200]. This anticancer activity is matched with pro-apoptotic, antiproliferative and immunomodulatory effects, which are considered necessary to reduce the disease. They are joined with treatment-related symptoms and in condition to improve the health-related quality of life.

Once the cytotoxic activity of microProteins, i.e., viscotoxins and viscolectins, was discovered, the problem of the mechanism of activity of *V. album* preparations was solved [79]. However, the belief that these peptides are unstable when ingested led to their administration by subcutaneous injection. The persistence of activity in oral prescriptions, as well their use in complementary medicine, changed some perspectives on this point. Thanks to new analytic methods based on hyphenated instrumentations, it is now evident that the proteome of *V. album* is much more complicated [201,202,203,204]. 

It is now possible to organize all of the aforementioned pieces of information to depict a coherent framework. *V. album*, in the form of hydro-alcoholic extract of foliage and fruits, can exert a selective cytotoxic activity against cancer cells, as evidenced by several studies. In particular, de Oliveira Mello, studying the phenolic compounds from *V. album* tinctures, which enhanced antitumor activity in melanoma murine cancer cells [205], investigated the toxicity of hydro-alcoholic extracts of *V. album* on different cell lines to assess their selectivity. The results are evident, since the control tinctures decreased the viability of B16F10 cells (murine melanoma) and the K562 (human chronic myelogenous leukemia cell line) in a dose-response way, where B16F10 presented a higher sensitivity. On the contrary, the MA-104 cell line, consisting of a normal cell line (monkey kidney, non-tumoral), resulted in resistance to all hydro-alcoholic concentrations used. The conclusion, on the basis of the reported data, is that *V. album* preparations obtained by hydro-alcoholic extraction of *V. album*, showed a selective tumor cytotoxicity with apoptosis induction and cell cycle effect. The next rational step consists of the detection of the substances responsible for the selective action, including microProteins and phenols.

Zarković et al. [206] reported that low concentrations of *V. album* commercial extracts (Isorel) inhibited B16F10 and HeLa tumor cell lines, and the observed effects resulted more strongly than purified lectin-1. Therefore, the authors considered that the therapeutic effect of the tested commercial preparation Isorel must be assigned to the association of low and high molecular weight components present in the extracts. Other authors share this interpretation. However, the general pharmacological idea, derived from the magic bullet axiom, is that one substance, or several substances that are structurally similar, must be investigated as responsible for a specific activity. The phytocomplex concept works with the opposite interpretation. Further confirmation about the bioactivity of other constituents came from the study of activity of flavonoid and phenolic acid constituents, which were examined by molecular docking and dynamic simulations and showed a stable interaction with target proteins and drug-likeness properties [207].

The study of de Mello also showed selective evident changes in the plasma membrane of the tumoral cell lines after treatment with the hydro-alcoholic extracts [208]. The plasma membrane is fundamental for the exchange of materials, as well the attack on the cell by external factors. This activity is derived from the capacity to selectively link to components of the membrane lipids, carbohydrates and proteins, which are characteristic constituents of cancer cells. 

If we assume that the *V. album* extracts display their action as a phytocomplex, wherein besides the microProteins such as viscotoxins and viscolectins, many other constituents can take part in the main property, including antioxidant, anti-inflammatory and anti-stress activity, this contributes to the general actions in restoring or maintaining homeostasis. The immunoadjuvanten and immunomodulatory properties fully confirm the capacities of *V. album*, in accordance with the ancient ethnobotanical indications and as in line with modern medical approaches [209,210,211,212,213,214,215,216].

Although a further study has confirmed the selective action of *V. album* extracts on cancer cells, leaving no effects on the normal ones [217] and fully confirming the observation of clinical trials about safety, as with any active substance, attention must concern about dosage [218,219]. As in any medicinal drug, the dose is a key factor. Concentrated products can cross the red line between benefit and damage. As a matter of fact, the number of scientific publications concerning *V. album* preparations increased exponentially in the last ten years, accordingly with interest in the therapeutic uses of derived products, which are widespread in food, food supplements and botanicals, with prevalence for phytomedicines in treatment of degenerative joint disease and as a palliative for malignant tumors in the form of extracts, such as Iscador, Isorel, Eurixor, Plenesol, Vysorel, Lektinol, Helixor, Cefalektin, ABNOVAviscum and Lektinol, again with positive results in safety and survival. Therefore, the concentration of active substances in these preparations must be considered safe, but there are also several concerns about the utilization in higher concentrations of some constituents, including microProteins. These compounds are known to produce dose-dependent hypertension or hypotension, bradycardia and increased uterine and gastrointestinal motility. However, the published literature on the human toxicity of mistletoe-containing products indicates that the majority of patients evidenced good tolerability and remained asymptomatic, with no reported deaths [111,129,130,220,221,222,223,224,225,226,227,228,229,230,231,232,233,234,235,236].

Furthermore, veterinary medicine and/or homeopathy are the most promising fields for future applications [237,238,239,240,241,242,243,244,245,246,247,248,249,250,251,252,253,254,255,256,257,258,259,260,261], not excluding the antiviral properties, which ask for further continuous attention [262]. Therefore, the knowledge about European mistletoe properties is in continuous evolution and requires a continuous revision of its use in evidence-based complementary and alternative medicine [263,264,265,266]. 

The absence of serious adverse effects is confirmed by hundreds of reports of pharmaco-phyto-vigilance, although in many cases there are local collateral effects, such as rush, itching, low fever and eczema when the drug is administrated by subcutaneous injection (i.e., Iscador e AbnobaVISCUM) [267,268,269,270,271]. 

The experiences obtained from the utilization of *V. album* extract as a complementary and adjuvant drug against cancer encourage its utilization in other fields, including the production of products that are viscotoxin-free but still useful against inflammation [272], which must be validated by further studies and research.

In conclusion, *V. album*, in the form of hydro-alcoholic extract of foliage and fruits, exerts a selective cytotoxic activity against cancer cells. The activity is derived from the capacity to selectively link to the membrane proteins, which have a characteristic constitution in cancer cells [273]. Once linked, the active substances should be able to interact with the metabolism of the cancer cells and also with the microenvironment surrounding the cells, which can be the cause of the disease but in any case is involved in its development, including the inflammation effects [274].

This suggests a selective additional possible mechanism in induced apoptosis, through the binding to certain cell receptors, as in some human galectins [275]. Galectins are a small family of proteins containing carbohydrate-binding proteins. Although only 15 members are known so far, they are involved in many widespread physiological processes, such as inflammation, immune responses, fibrosis, autophagy, signalling and heart disease, as well as progression of metastasis of cancer. In addition to the aforementioned viscumin [276,277,278,279], more attention could be focused on other viscolectins, such as *Viscum album* agglutinin (VAA), which exerts various biological effects along with the cytotoxic properties for tumor cells in culture [142,171,280]. The activity of VAA was considered similar to galectin-1 [281,282]. Galectins, which are extracellular endogenous lectins, possess galactose-specific surface-binding sites. Galectins are well studied glycan-binding proteins, which mediate multiple biological functions, including immunity and anti-inflammatory effects, and have been shown to stimulate natural killer cells, granulocytes [283]. Noncytotoxic concentrations of VAA-I enhanced several anti-inflammatory effects, such as secretion of pro-inflammatory cytokines in cultures of human peripheral blood mononuclear cells, peripheral blood lymphocytes (PBL), human peripheral blood monocytes (PBM), murine thymocytes and human monocytic, and in an animal model VAA-I [284,285].

The *V. album* extracts display their action as phytocomplex, wherein besides the microProteins such as viscotoxins and viscolectins, they act with extracellular signal-regulated enzymes [286,287], as well many other constituents that can take part in the main property, antioxidant, anti-inflammatory and anti-stress activity, which contributes to the general actions in restoring or maintaining homeostasis. Particular attention should be concentrated on the constituents of the metabolome, including the discovery of new active constituents that are very different from microProteins [288]. The experiences regarding the utilization of European mistletoe extracts as a complementary and adjuvant drug against cancer encourage its utilization in other fields, which must be validated by further studies and research on the properties of this very special plant, as evidenced by recent publications [50,289].

## Figures and Tables

**Table 1:** Reported anti-inflammatory activities of extracts of European mistletoe (VA) and Korean mistletoe.

Part of the Plant	Reported Activity	Active Constituents	Reference
VA hydro-alcoholic extract	Anti-inflammatory effect by selectively inhibiting cytokine-induced expression of cyclooxygenase-2	Total extract	[135]
VA hydro-alcoholic extract	Inhibition of cytokine-induced PGE2 via selective inhibition of COX-2 and destabilization of COX-2 mRNA	Total extract	[136]
Aqueous extract	Proliferation and cytokine synthesis of splenocytes	Total extract	[137]
VA hydro-alcoholic extract	Destabilization of COX-2 mRNA	Total extract	[138]
KV mistletoe extract	Increase in the levels of serum inflammatory cytokines	Total extract	[139]
VA ethanolic extract of leaves and twigs	Anti-inflammatory activity	Free and glycoside flavonoids	[141]
VA hydro-alcoholic extract	Induction apoptosis of preactivated neutrophils in vivo without pro-inflammatory response	Agglutinin-I (VAA-I)	[142]
VA hydro-alcoholic extract	eEffect on human peripheral blood lymphocytes	Agglutinin-I (VAA-I)	[171]

**Table 2:** Reported anti-oxidation activities of extracts of Viscum album (VA).

Part of the Plant	Reported Activity	Active Constituents	Reference
VA extract of leaves and stems	Antioxidant activity	Total phenols	[143]
VA methanolic extract	Inhibitory effect on lipid peroxidation	Total extract	[144]
VA methanolic extract	Antiproliferative and antioxidant properties in vitro	Methanolic extract	[145]
VA ethanolic extract	Increased percentage of low plasma antioxidant activity	eEhanolic extract	[146]
VA methanolic extract	Antioxidant activity and scavenging activity	Methanolic extract	[147]
VA aqueous extracts	Antioxidant and anti-inflammatory activities	Phenolic acids, phenylpropanoids and flavonoids	[148]
VA aqueous extracts	Antioxidant activity	Polyphenols and flavonoids	[149]
VA alcoholic extracts	Antioxidant activity and scavenging activity	Polyphenols and flavonoids	[150]
VA aqueous and ethanolic extracts	Antioxidant activity	Total extract	[155]
VA aqueous extract of leaves	Antioxidant activity	Total extract	[156]
VA aqueous and methanolic extracts	Antioxidant activity	Total extracts and phenols	[158]
VA crude extract	Antioxidant activity	Phenols	[159]
KV mistletoe extract	Overexpressions of cyclooxygenase-2 and inducible NO synthase	Lectins	[141]
KV extract	Antioxidant activity	Caffeic acid and lyoniresinol	[165]
VA ethanolic extract	Inhibition of ADP-induced platelet aggregation	Phenylpropanoid glycosides	[166]
VA extract of leaves	Antioxidant activity	Ramnazin glucosides	[167]
Methanol extract of whole shrub	Antioxidant activity	Phenols	[168]
VA methanol extract aerial parts	Antioxidant activity	Total extract	[172]

## Data Availability

No new data were created.
